# Supplementary material for: Disentangling ERBB Signaling in Breast Cancer Subtypes—A Model-Based Analysis
Source: Cancers (Basel). 2022 May 12;14(10):2379. doi: 10.3390/cancers14102379 (PMC9139462; doi:10.3390/cancers14102379)
Supplement: Supplementary file 1 [file cancers-14-02379-s001.zip › cancers-1684785-supplementary/Supplement.pdf]

Supplementary Materials: Disentangling ERBB Signaling in Breast Cancer Subtypes – a Model-Based Analysis

Svenja Kemmer, Mireia Berdiel-Acer, Eileen Reinz, Johanna Sonntag, Nooraldeen Tarade, Stephan Bernhardt, Mirjam Fehling-Kaschek, Max Hasmann, Ulrike Korf, Stefan Wiemann and Jens Timmer

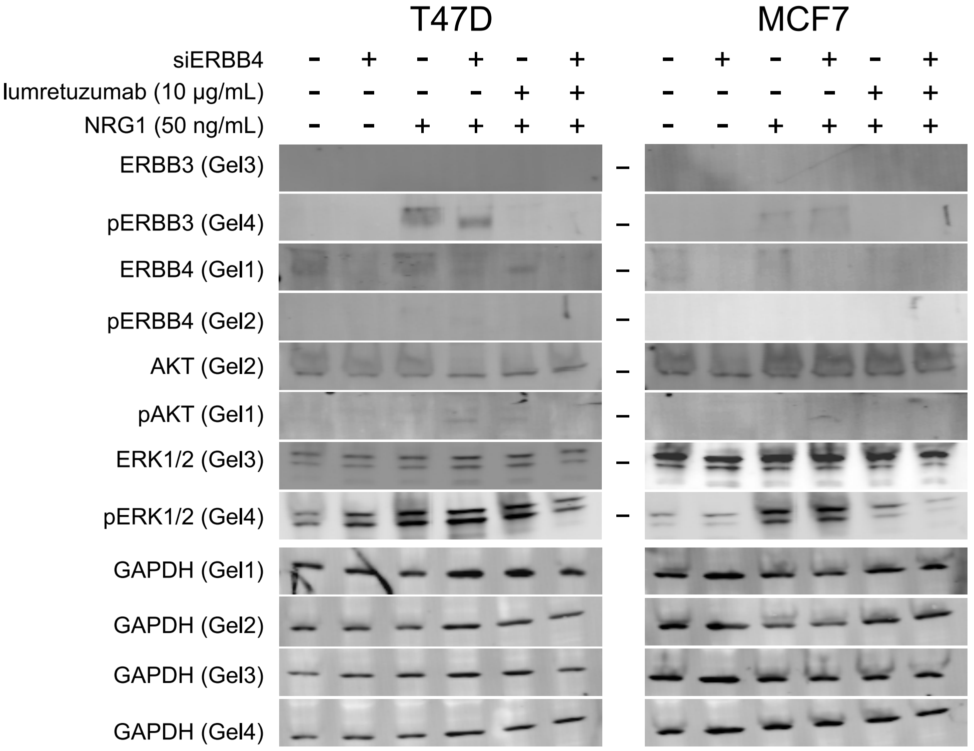

Figure S1. NRG1 induces signaling via ERBB3, and not ERBB4. *ERBB4* was (+) or not (-) knocked down in T47D and MCF7 cell lines, and alterations in total levels and phosphorylation of ERBB3 and ERBB4 receptors as well as of downstream targets AKT and ERK1/2 assessed. Cells were additionally treated with lumretuzumab and/or NRG1 to block and induce ERBB3 signaling, respectively.

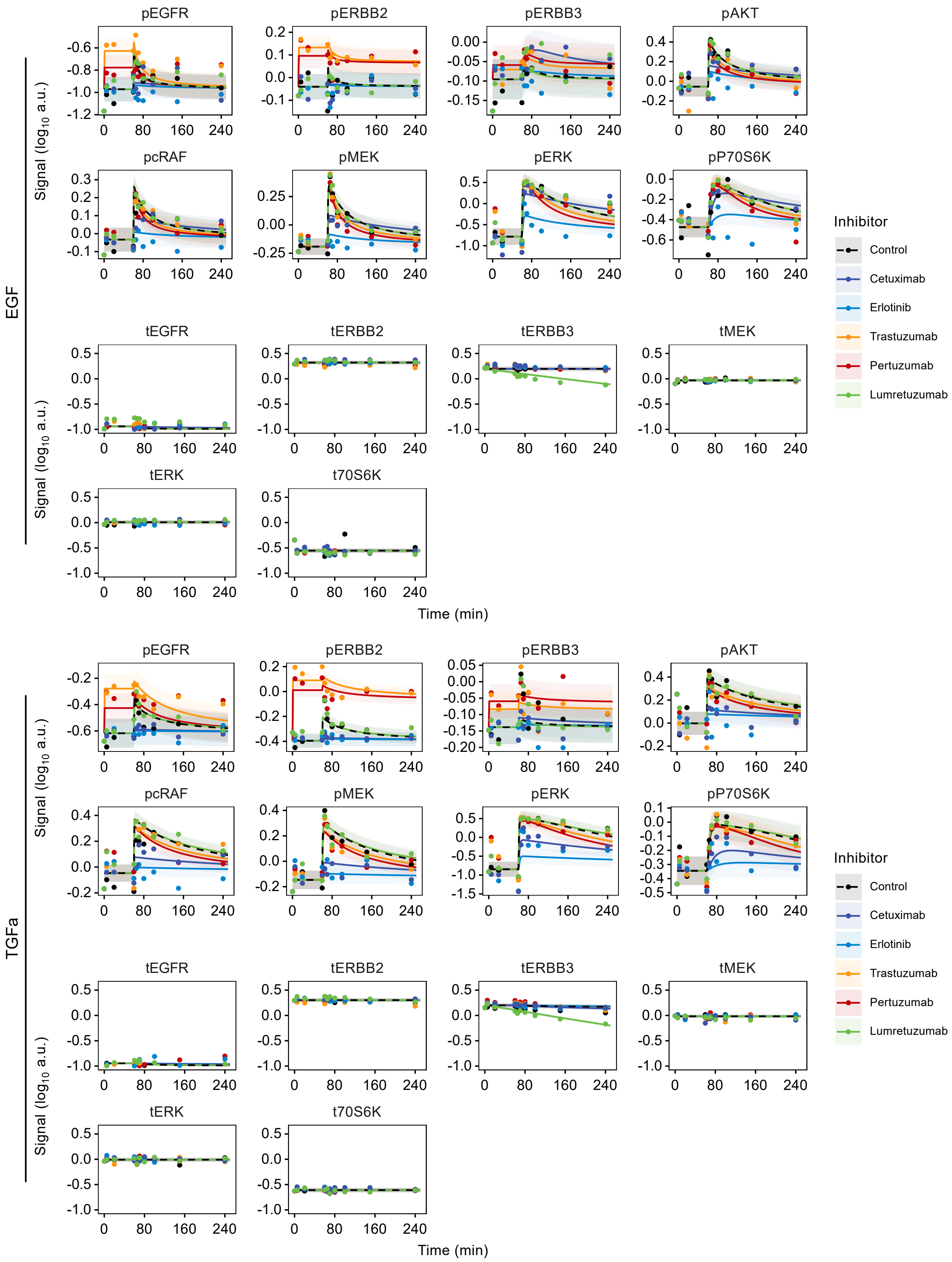

Figure S2. RPPA measurements and model fits of T47D cells.

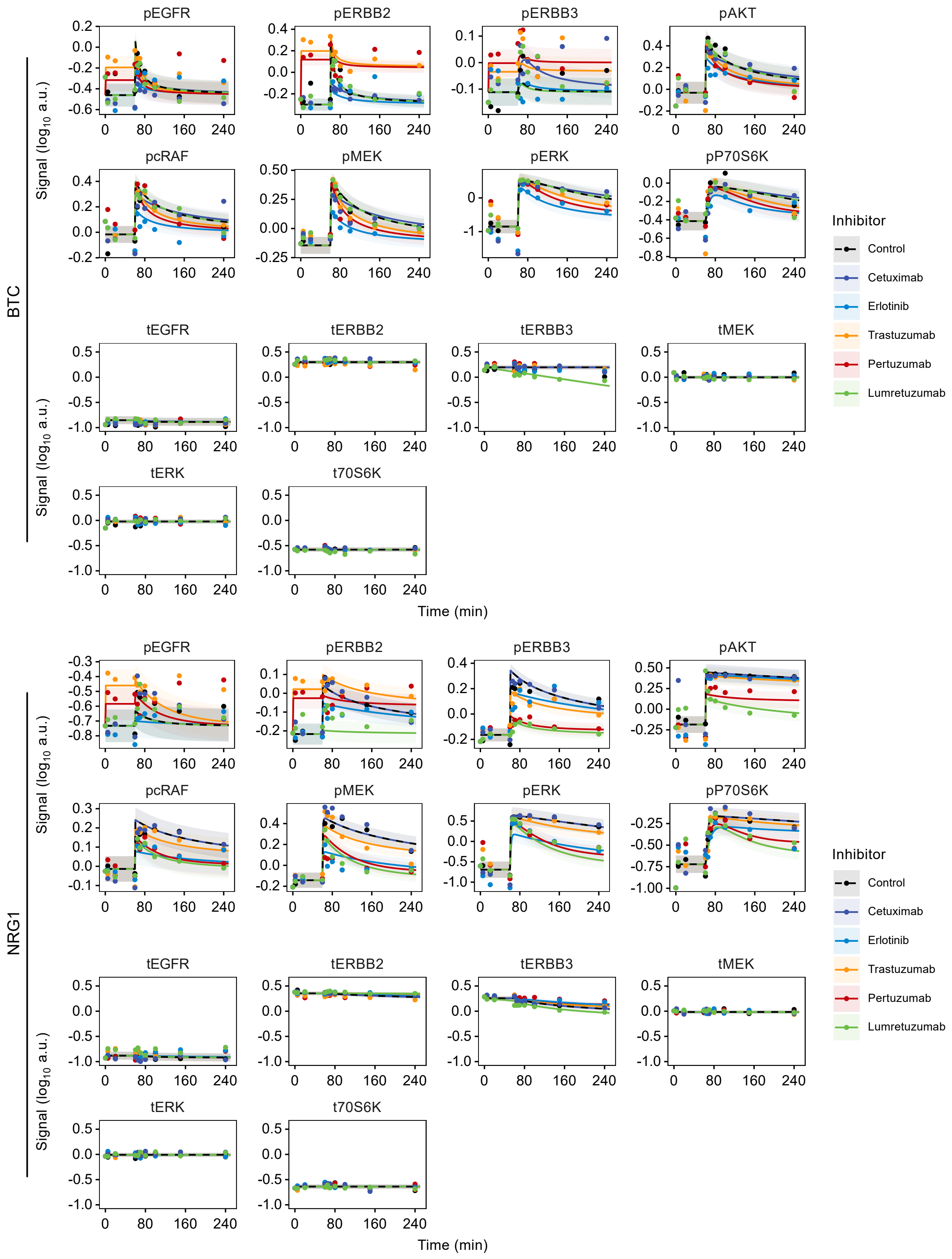

Figure S2. (continued) RPPA measurements and model fits of T47D cells. The cellular response to the different drugs is depicted under stimulation with EGF, TGFa, BTC or NRG1. Data represents the mean of biological replicates (n=3) and is displayed as dots. Trajectories of the model fit are indicated as solid lines.

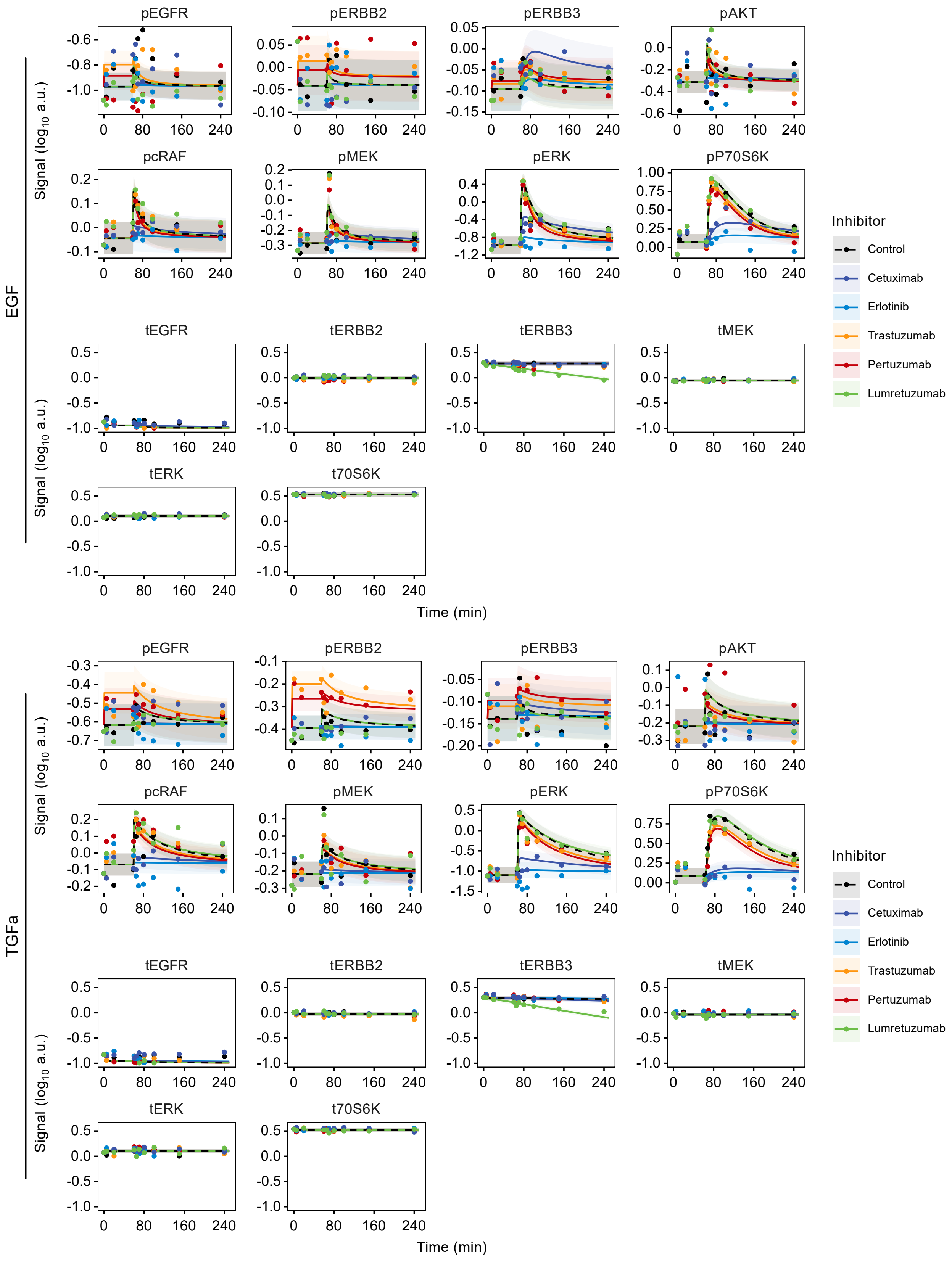

Figure S3. RPPA measurements and model fits of MCF7 cells.

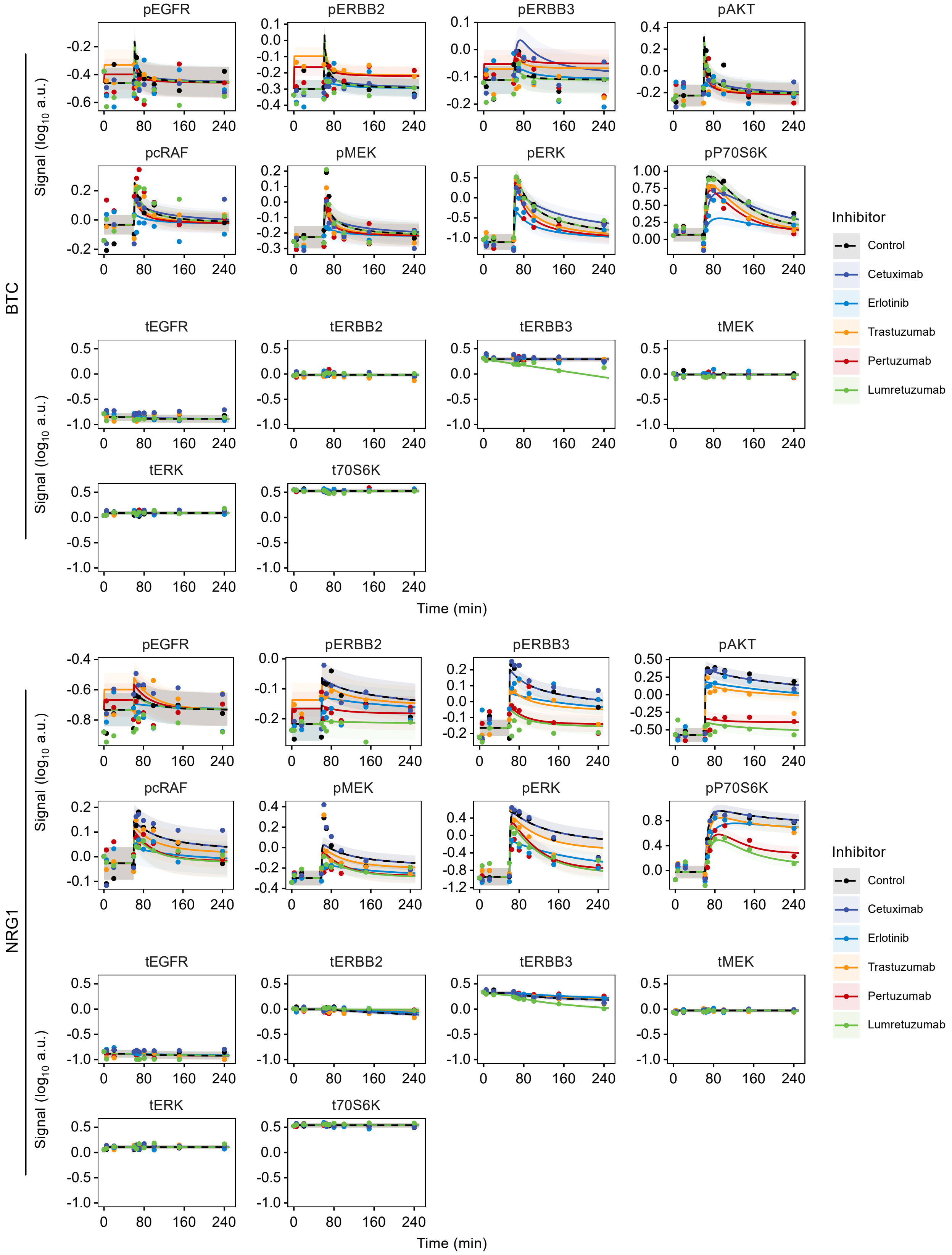

Figure S3. (continued) RPPA measurements and model fits of MCF7 cells. The cellular response to the different drugs is depicted under stimulation with EGF, TGFa, BTC or NRG1. Data represents the mean of biological replicates (n=3) and is displayed as dots. Trajectories of the model fit are indicated as solid lines.

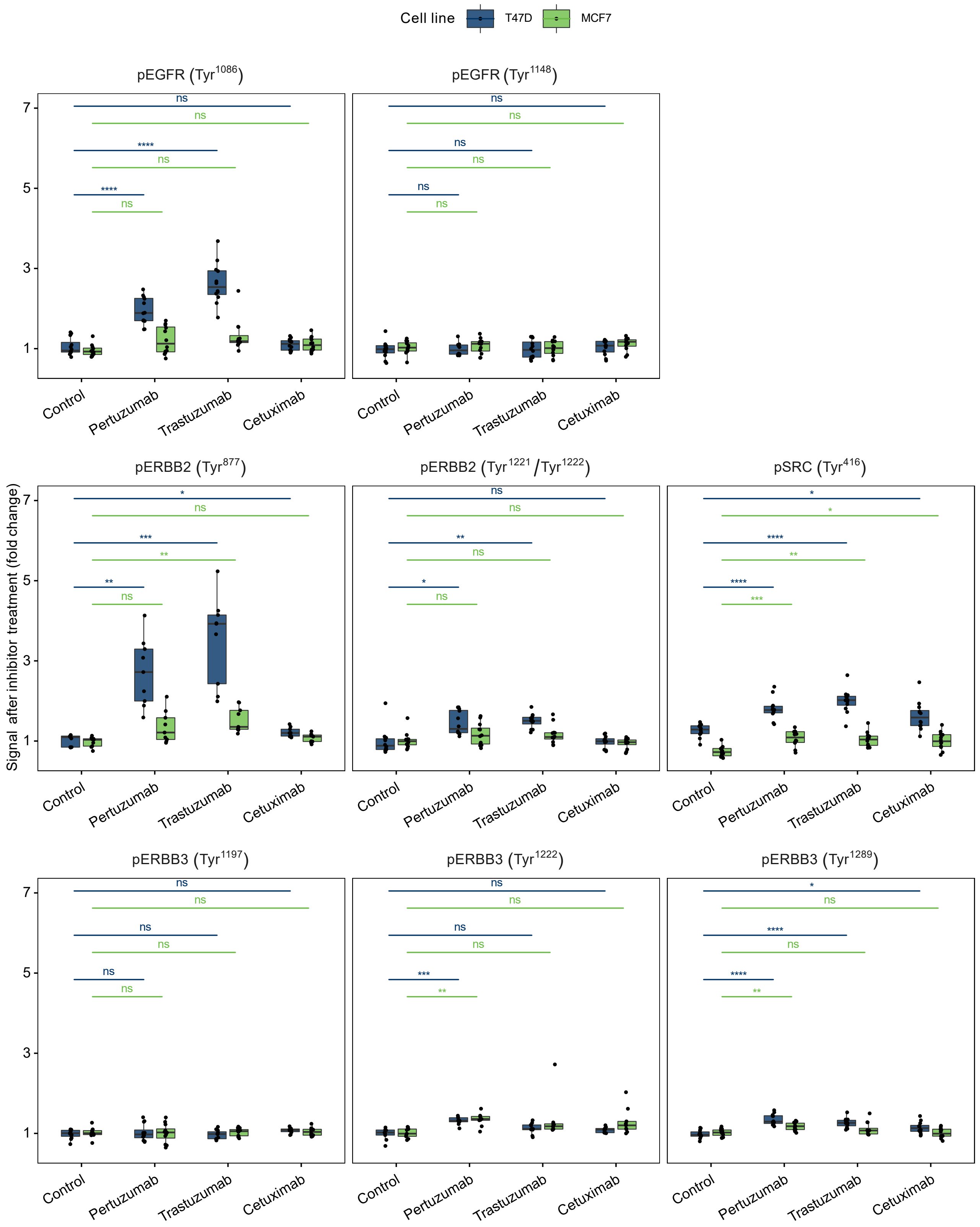

Figure S4. Antibody-induced phosphorylation of ERBB receptors in T47D and MCF7. The protein phosphorylation of EGFR, ERBB2 and ERBB3 and SRC at indicated sites are displayed for T47D and MCF7. Cells were treated with pertuzumab, trastuzumab or cetuximab or were left untreated for five minutes until the phosphorylation level was quantified by RPPA. Each condition is represented by 9-12 replicates. Significance levels indicate adjusted p values with the cutoffs: p-value.adj < 1 = n.s., < 0.05 = *, < 0.01 = **, < 0.001 = ***, < 1e-04 = ****.

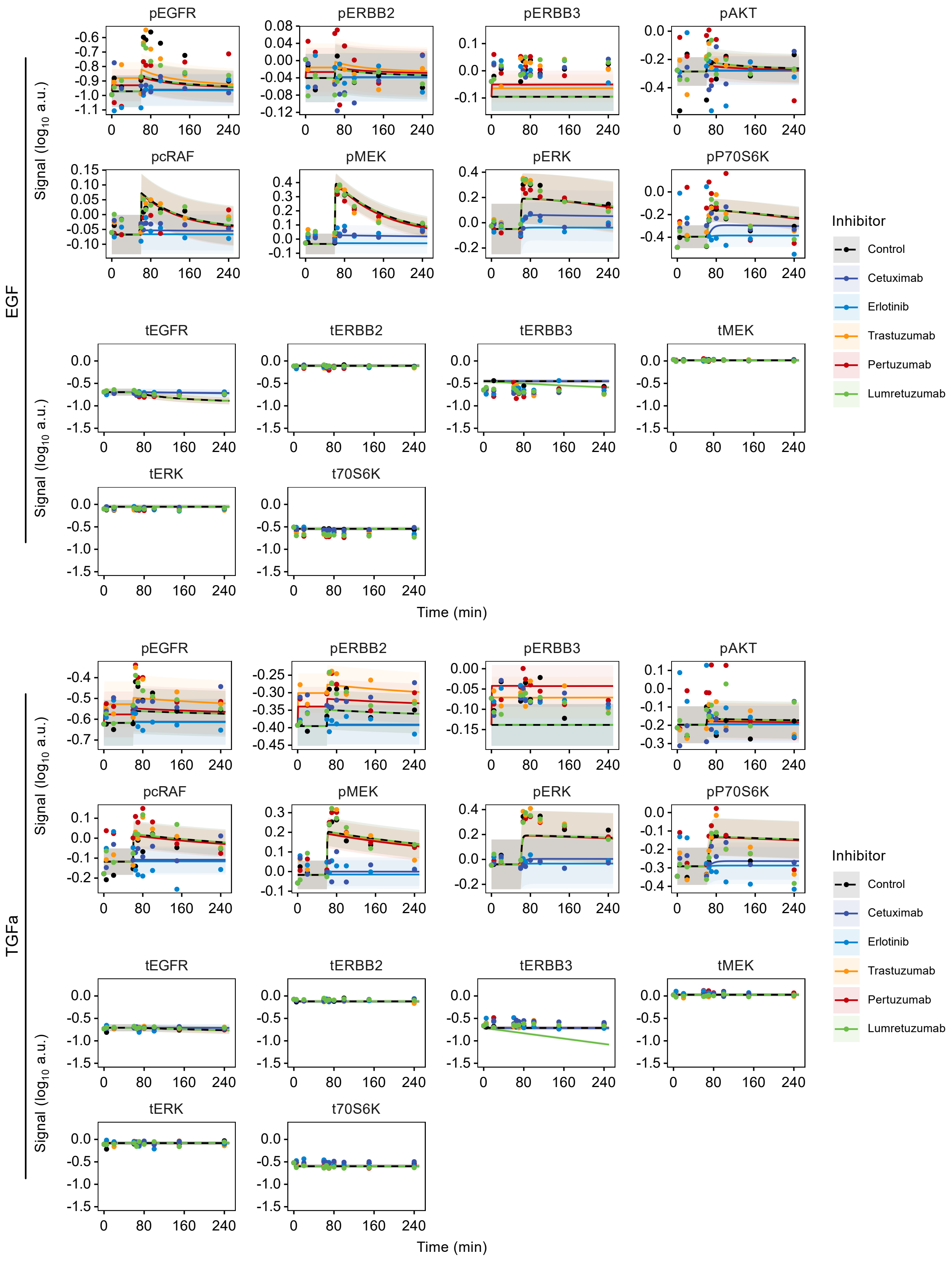

Figure S5. RPPA measurements and model fits of MDA-MB-231 cells.

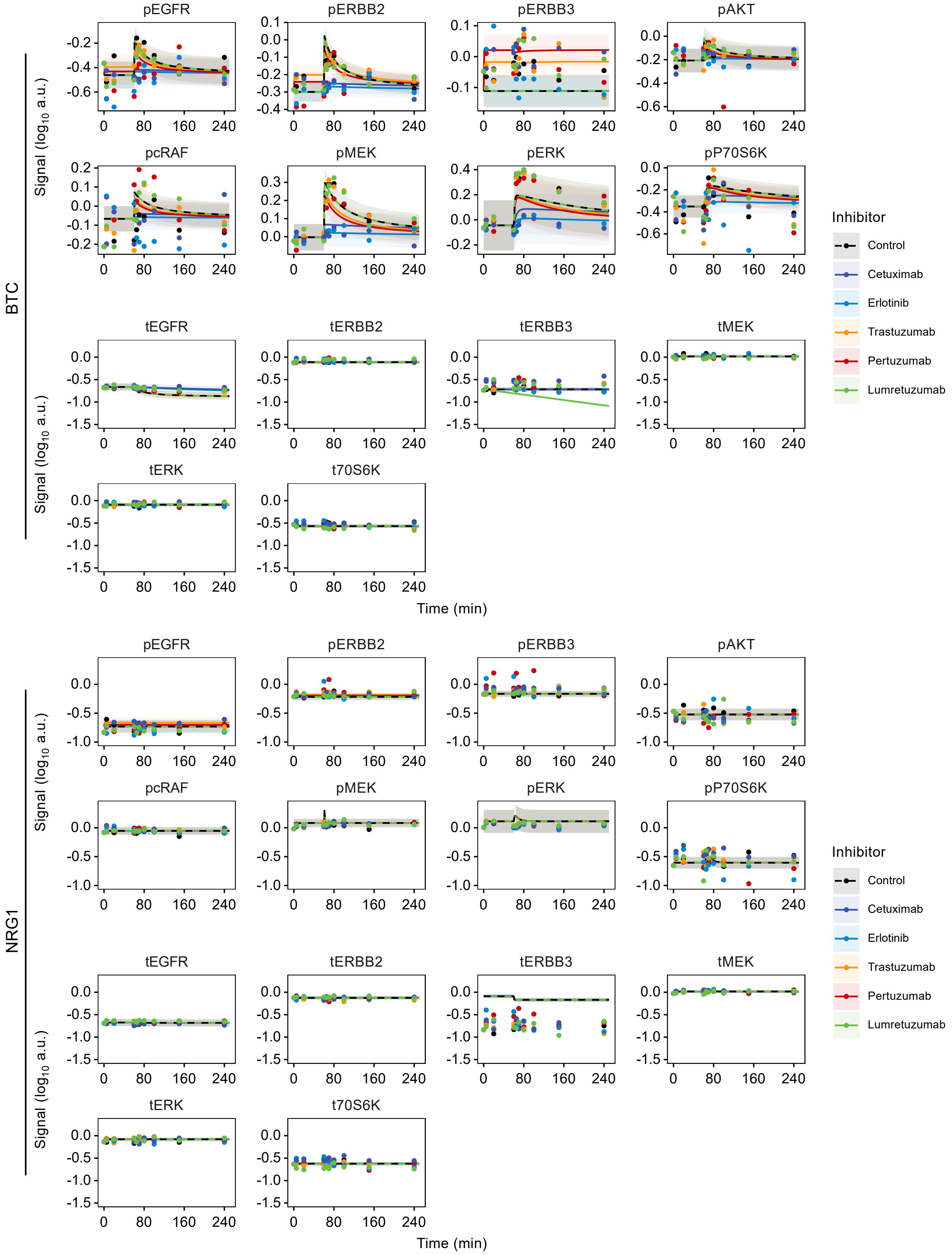

Figure S5. (continued) RPPA measurements and model fits of MDA-MB-231 cells. The cellular response to the different drugs is depicted under stimulation with EGF, TGFa, BTC or NRG1. Data represents the mean of biological replicates (n=3) and is displayed as dots. Trajectories of the model fit are indicated as solid lines.

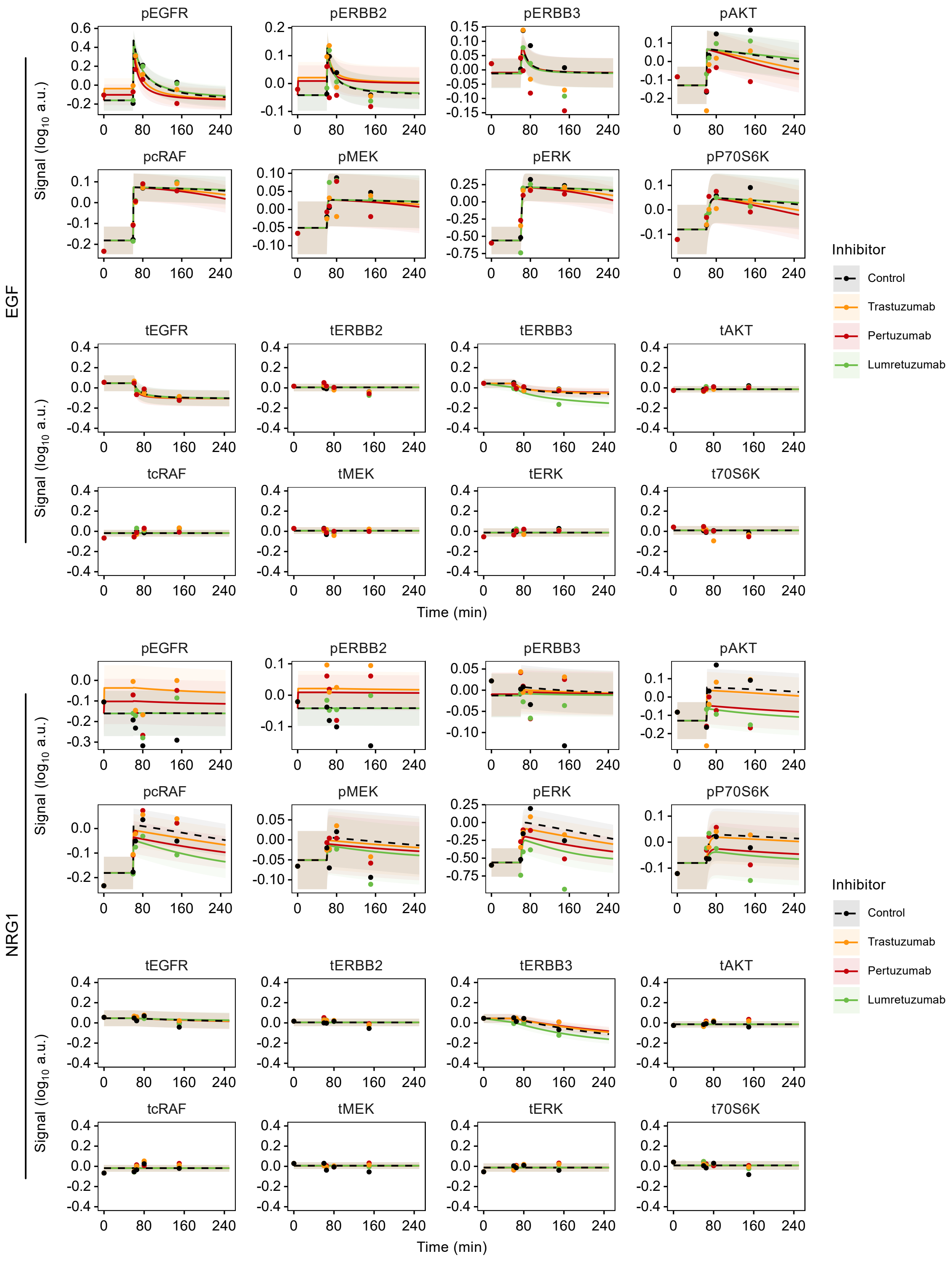

Figure S6. RPPA measurements and model fits of SKBR3 cells. The cellular response to the different drugs is depicted under stimulation with EGF or NRG1. Data represents the mean of biological replicates (n=3) and is displayed as dots. Trajectories of the model fit are indicated as solid lines.

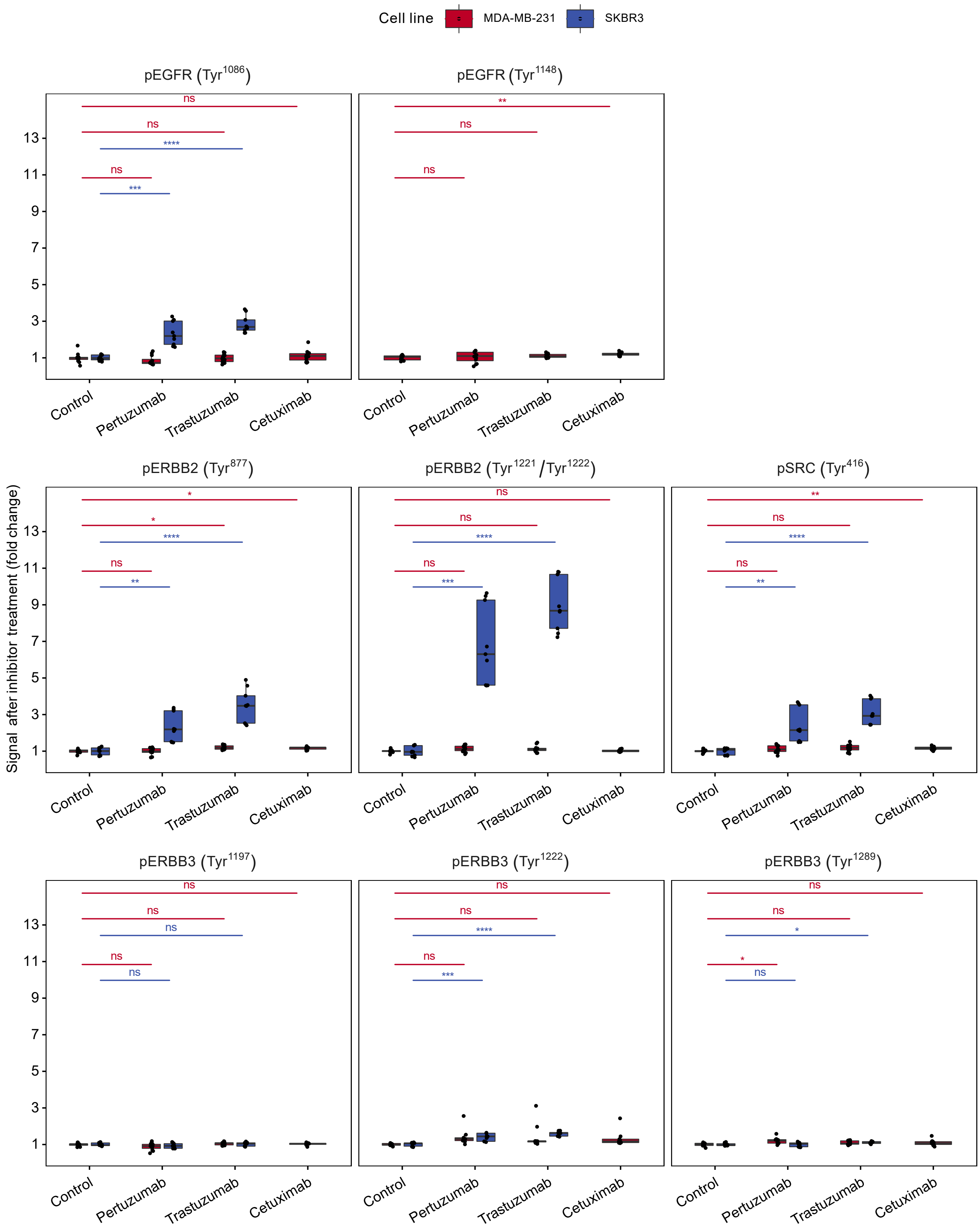

Figure S7. Antibody-induced phosphorylation of ERBB receptors in MDA-MB-231 and SKBR3. The protein phosphorylation of EGFR, ERBB2 and ERBB3 and SRC at indicated sites are displayed for MDA-MB-231 and SKBR3. Cells were treated with pertuzumab, trastuzumab or cetuximab or were left untreated for five minutes until the phosphorylation level was quantified by RPPA. Each condition is represented by 9-12 replicates. Significance levels indicate adjusted p values with the cutoffs: p-value.adj < 1 = n.s., < 0.05 = *, < 0.01 = **, < 0.001 = ***, < 1e-04 = ****.

Table S1. Primary antibodies.

Product Name	Target Protein	UniProt ID	Gene Symbol	Specificity	Vendor	Antibody ID	Host
EGF Receptor (C74B9) Rabbit mAb	EGFR	P00533	*EGFR*	total	CST	2646	rabbit
EGF Receptor Antibody	EGFR	P00533	*EGFR*	total	CST	2232	rabbit
Phospho-EGF Receptor (Tyr^{1086}) Antibody	EGFR	P00533	*EGFR*	$pTyr^{1086}$	CST	2220	rabbit
Phospho-EGF Receptor (Tyr^{1148}) Antibody	EGFR	P00533	*EGFR*	$pTyr^{1148}$	CST	4404	rabbit
Phospho-HER4/ErbB4 (Tyr^{1284})/EGFR (Tyr^{1173}) (21A9) Rabbit mAb	ERBB4	P00533	*EGFR*	$pTyr^{1173}$	CST	4757	rabbit
Epredia™ HER-2/c-erbB-2/neu Ab-17, mAb	ERBB2	P04626	*ERBB2*	total	Fisher scientific	12612187	mouse
Phospho-HER2/ErbB2 (Tyr^{877}) Antibody	ERBB2	P04626	*ERBB2*	$pTyr^{877}$	CST	2241	rabbit
Phospho-HER2/ErbB2 ($Tyr^{1221}Tyr^{1222}$) (6B12) Rabbit mAb	ERBB2	P04626	*ERBB2*	$pTyr^{1221}pTyr^{1222}$	CST	2243	rabbit
HER3/ErbB3 (D22C5) XP® Rabbit mAb	ERBB3	P21860	*ERBB3*	total	CST	12708	rabbit
Anti-erbB-3/HER-3 Antibody, clone 2F12-AB2	ERBB3	P21860	*ERBB3*	total	Merck	05-390	mouse
Phospho-HER3/ErbB3 (Tyr^{1197}) (C56E4) Rabbit mAb	ERBB3	P21860	*ERBB3*	$pTyr^{1197}$	CST	4561	rabbit
Phospho-HER3/ErbB3 (Tyr^{1222}) (50C2) Rabbit mAb	ERBB3	P21860	*ERBB3*	$pTyr^{1222}$	CST	4784	rabbit
Phospho-HER3/ErbB3 (Tyr^{1289}) (21D3) Rabbit mAb	ERBB3	P21860	*ERBB3*	$pTyr^{1289}$	CST	4791	rabbit
Recombinant Anti-ErbB4 / HER4 antibody [EP192Y]	ERBB4	Q15303	*ERBB4*	total	Abcam	ab76303	rabbit
Recombinant Anti-ErbB4 / HER4 (Tyr^{1162}) antibody [EP2270Y]	ERBB4	Q15303	*ERBB4*	$pTyr^{1162}$	Abcam	ab68478	rabbit
Phospho-HER4/ErbB4 (Tyr^{1284})/EGFR (Tyr^{1173}) (21A9) Rabbit mAb	ERBB4	Q15303	*ERBB4*	$pTyr^{1284}$	CST	4757	rabbit
c-Raf Antibody	cRAF	P04049	*RAF1*	total	CST	9422	rabbit
Phospho-c-Raf ($Ser^{289}Ser^{296}Ser^{301}$) Antibody	cRAF	P04049	*RAF1*	$pSer^{289}pSer^{296}pSer^{301}$	CST	9431	rabbit
Phospho-c-Raf (Ser^{338}) (56A6) Rabbit mAb	cRAF	P04049	*RAF1*	$pSer^{338}$	CST	9427	rabbit
Phospho-c-Raf (Ser^{259}) Antibody	cRAF	P04049	*RAF1*	$pSer^{259}$	CST	9421	rabbit
Purified Mouse Anti-MEK1 Clone 25/MEK1 (RUO)	MEK1	Q02750	*MAP2K1*	total	BD Biosciences	610121	mouse
Phospho-MEK1/2 ($Ser^{217}Ser^{221}$) (41G9) Rabbit mAb	MEK1	Q02750	*MAP2K1*	$pSer^{217}pSer^{221}$	CST	9154	rabbit
Anti-phospho-MAP Kinase Kinase 1/2 ($Ser^{217}Ser^{221}$) antibody produced in rabbit	MEK1	Q02750	*MAP2K1*	$pSer^{217}pSer^{221}$	Sigma	M7683	rabbit
Phospho-MEK1/2 ($Ser^{217}Ser^{221}$) (41G9) Rabbit mAb	MEK2	P36507	*MAP2K2*	$pSer^{222}pSer^{226}$	CST	9154	rabbit
p44/42 MAPK (Erk1/2) Antibody	ERK1	P27361	*MAPK3*	total	CST	9102	rabbit
p44/42 MAPK (Erk1/2) Antibody	ERK2	P28482	*MAPK1*	total	CST	9102	rabbit
Phospho-p44/42 MAPK (Erk1/2) ($Thr^{202}Tyr^{204}$) (D13.14.4E) XP® Rabbit mAb	ERK1	P27361	*MAPK3*	$pThr^{202}pTyr^{204}$	CST	4370	rabbit

Product Name	Target Protein	UniProt ID	Gene Symbol	Specificity	Vendor	Antibody ID	Host
Phospho-p44/42 MAPK (Erk1/2) (Thr^{202}Tyr^{204}) (D13.14.4E) XP® Rabbit mAb	ERK2	P28482	*MAPK1*	pThr^{185}pTyr^{187}	CST	4370	rabbit
Akt (pan) (C67E7) Rabbit mAb	AKT1	P31749	*AKT1*	total	CST	4691	rabbit
Akt (pan) (C67E7) Rabbit mAb	AKT2	P31751	*AKT2*	total	CST	4691	rabbit
Akt (pan) (C67E7) Rabbit mAb	AKT3	Q9Y243	*AKT3*	total	CST	4691	rabbit
A(610860) BD Transduction Laboratories™ Purified Mouse anti-Akt	AKT1	P31749	*AKT1*	total	BD Biosciences	610860	rabbit
(610860) BD Transduction Laboratories™ Purified Mouse anti-Akt	AKT2	P31751	*AKT2*	total	BD Biosciences	610860	rabbit
(610860) BD Transduction Laboratories™ Purified Mouse anti-Akt	AKT3	Q9Y243	*AKT3*	total	BD Biosciences	610860	rabbit
Phospho-Akt (Thr^{308}) Antibody	AKT1	P31749	*AKT1*	pThr^{308}	CST	9275	mouse
Phospho-Akt (Thr^{308}) Antibody	AKT2	P31751	*AKT2*	pThr^{309}	CST	9275	mouse
Phospho-Akt (Thr^{308}) Antibody	AKT3	Q9Y243	*AKT3*	pThr^{305}	CST	9275	mouse
Phospho-Akt (Ser^{473}) Antibody	AKT1	P31749	*AKT1*	pSer^{473}	CST	9271	rabbit
Phospho-Akt (Ser^{473}) Antibody	AKT2	P31751	*AKT2*	pSer^{474}	CST	9271	rabbit
Phospho-Akt (Ser^{473}) Antibody	AKT3	Q9Y243	*AKT3*	pSer^{472}	CST	9271	rabbit
p70 S6 Kinase (49D7) Rabbit mAb	p70S6Kinase	P23443	*RPS6KB1*	total	CST	2708	rabbit
Phospho-p70 S6 Kinase (Thr^{389}) (108D2) Rabbit mAb	P70S6Kinase	P23443	*RPS6KB1*	pThr^{389}	CST	9234	rabbit
Phospho-Src Family (Tyr^{416}) Antibody	SRC	P12931	*SRC*	pY^{419}	CST	2101	rabbit
Phospho-Src Family (Tyr^{416}) Antibody	Yes	P07947	*Yes*	pY^{426}	CST	2101	rabbit
Phospho-Src Family (Tyr^{416}) Antibody	LCK	P06239	*LCK*	pY^{394}	CST	2101	rabbit
Phospho-Src Family (Tyr^{416}) Antibody	LYN	P07948	*LYN*	pY^{397}	CST	2101	rabbit
GAPDH antibody [GT239]	GAPDH	P04406	*GAPDH*	total	GeneTex	GTX627408	mouse

Table S2. Secondary antibodies.

Product Name	Target Protein	Fluorophore	Vendor	Antibody ID	Host
F(ab')2-Goat anti-Rabbit IgG (H+L) Cross-Adsorbed Secondary Antibody, Alexa Fluor 680	rabbit	Alexa680	Life Technologies	A21077	goat
F(ab')2-Goat anti-Mouse IgG (H+L) Secondary Antibody, Alexa Fluor® 680 conjugate	mouse	Alexa680	Life Technologies	A21059	goat
Goat anti-Mouse IgG (H+L) Secondary Antibody, DyLight™ 800 4X PEG	mouse	DyLight™ 800 4X PEG	Thermo Fisher	SA5-35521	goat

Table S3. Therapeutic antibodies, kinase inhibitors and ligands.

**Therapeutic antibodies targeting receptor tyrosine kinases**			
**Product Name**	**Target Protein**	**Drug Name**	**Vendor**
cetuximab	EGFR	Erbitux	Merck KGaA
trastuzumab	ERBB2	Herceptin	Roche
pertuzumab	ERBB2	Perjeta	Roche
lumretuzumab	ERBB3	RG7116	Roche

**Receptor tyrosine kinase inhibitor**			
**Product Name**	**Target Protein**	**Drug Name**	**Vendor**
erlotinib	EGFR	Tarceva	Roche

**Ligands**					
**Product Name**	**Protein Name**	**UniProt ID**	**Gene Symbol**	**Vendor**	**Product ID**
EGF, recombinant, expressed in E. coli, lyophilized powder, suitable for cell culture	EGF	P01133	*EGF*	Sigma Aldrich	E9644
Recombinant Human Betacellulin Protein	BTC	P35070	*BTC*	R&D Systems	261-CE/CF
Neuregulin/Heregulin-1β (NRG-1β/HRG-1β), human recombinant	NRG1/HRG1	Q02297	*NRG1*	BioVision	4711
Recombinant Human Amphiregulin Protein	AREG	P15514	*AREG*	R&D Systems	262-AR/CF
Transforming Growth Factor-α human	TGF-a	P01135	*TGFA*	Sigma Aldrich	T7924

Table S4. Model reactions. Each row corresponds to a molecular reaction as indicated by the model structure depicted in Figure 2. *Ligand* and *drug* are representatives for the individual ligand and drug concentrations. *(NISS)* indicates that corresponding reactions do not contribute to the steady state.

Educt	Product	Rate	Description
2 · R1	R1R1	build_R1R1 · (1/(1+drug_R1R1)) · R1 · R1 · ligand	R1R1 dimerization (NISS)
R1R1	2 · R1	decay_R1R1 · R1R1	R1R1 decay
R1 + R2	R1R2	build_R1R2 · (1/(1+drug_R1R2)) · R1 · R2 · ligand	R1R2 dimerization (NISS)
R1R2	R1 + R2	decay_R1R2 · R1R2	R1R2 decay
R1 + R3	R1R3	build_R1R3 · (1/(1+drug_R1R3)) · R1 · R3 · ligand	R1R3 dimerization (NISS)
R1R3	R1 + R3	decay_R1R3 · R1R3	R1R3 decay
R2 + R3	R2R3	build_R2R3 · (1/(1+drug_R2R3)) · R2 · R3 · ligand	R2R3 dimerization (NISS)
R2R3	R2 + R3	decay_R2R3 · R2R3	R2R3 decay
R1 + R2	R1R2_off	build_R1R2off · R1 · R2 · drug	R1R2 dimerization by tras/pert (NISS)
R1R2_off	R1 + R2	decay_R1R2off · R1R2_off	R1R2off decay
R2 + R3	R2R3_off	build_R2R3off · R2 · R3 · drug	R2R3 dimerization by tras/pert (NISS)
R2R3_off	R2 + R3	decay_R2R3off · R2R3_off	R2R3_off decay
2 · R2	R2R2_off	build_R2R2off · R2 · R2 · drug	R2R2 dimerization by tras/pert (NISS)
R2R2_off	2 · R2	decay_R2R2off · R2R2_off	R2R2_off decay
R1R1		degrade_R1R1 · R1R1	R1R1 degradation (NISS)
R1R2		degrade_R1R2 · R1R2	R1R2 degradation (NISS)
R1R3		degrade_R1R3 · R1R3	R1R3 degradation (NISS)
R2R3		degrade_R2R3 · R2R3	R2R3 degradation (NISS)
R3		degrade_R3_lumretuzumab · R3 · drug	R3 degradation by lum (NISS)
AKT	pAKT	phospho_AKT_R1R1 · AKT · R1R1	AKT to pAKT by R1R1 (NISS)
AKT	pAKT	phospho_AKT_R1R2 · AKT · R1R2	AKT to pAKT by R1R2 (NISS)
AKT	pAKT	phospho_AKT_R1R3 · AKT · R1R3	AKT to pAKT by R1R3 (NISS)
AKT	pAKT	phospho_AKT_R2R3 · AKT · R2R3	AKT to pAKT by R2R3 (NISS)
AKT	pAKT	phospho_AKT_basal · AKT	basal AKT to pAKT
pAKT	AKT	dephospho_pAKT · pAKT · mutation	pAKT to AKT
cRAF	pcRAF	phospho_cRAF_R1R1 · (1/(1+drug_cRAF_R1R1)) · cRAF · R1R1	cRAF activation by R1R1 (NISS)
cRAF	pcRAF	phospho_cRAF_R1R2 · (1/(1+drug_cRAF_R1R2)) · cRAF · R1R2	cRAF activation by R1R2 (NISS)
cRAF	pcRAF	phospho_cRAF_R1R3 · (1/(1+drug_cRAF_R1R3)) · cRAF · R1R3	cRAF activation by R1R3 (NISS)
cRAF	pcRAF	phospho_cRAF_R2R3 · (1/(1+drug_cRAF_R2R3)) · cRAF · R2R3	cRAF activation by R2R3 (NISS)
cRAF	pcRAF	phospho_cRAF_basal · cRAF	basal cRAF activation
pcRAF	cRAF	pcRAF · deact_cRAF	cRAF inactivation
MEK	pMEK	phospho_MEK · MEK · pcRAF	MEK to pMEK
pMEK	ppMEK	phospho_MEK · pMEK · pcRAF	pMEK to ppMEK
MEK	pMEK	phospho_MEK_basal · MEK	basal MEK to pMEK
pMEK	ppMEK	phospho_MEK_basal · pMEK	basal pMEK to ppMEK
ppMEK	pMEK	dephospho_pMEK · ppMEK	ppMEK to pMEK
pMEK	MEK	dephospho_pMEK · pMEK	pMEK to MEK
ERK	pERK	phospho_ERK · ERK · ppMEK	ERK to pERK
pERK	ppERK	phospho_ERK · pERK · ppMEK	pERK to ppERK
ppERK	pERK	dephospho_pERK · ppERK	ppERK to pERK
pERK	ERK	dephospho_pERK · pERK	pERK to ERK
S6K	pPS6K	phospho_S6K_AKT · S6K · pAKT	S6K to pPS6K by pAKT
S6K	pPS6K	phospho_S6K_ERK · S6K · ppERK	S6K to pPS6K by ppERK
pPS6K	S6K	dephospho_S6K · pPS6K	pPS6K to S6K

Table S5. Observables. Observables were computed with respect to model states as indicated. Measured components indicated with * and corresponding fixed offsets are taken vom Niepel *et al.* [1].

Measured component	Observable function
pEGFR_obs	log10(scale_pR1 · (2 · R1R1 + R1R2 + R1R3 + R1R2_off) + offset_pR1)
pERBB2_obs	log10(scale_pR2 · (R1R2 + R2R3 + R1R2_off + R2R3_off + 2 · R2R2_off) + offset_pR2)
pERBB3_obs	log10(scale_pR3 · (R1R3 + R2R3 + R2R3_off) + offset_pR3)
pAKT_obs	log10(scale_pAkt · pAKT + offset_pAkt)
pcRAF_obs	log10(scale_pcRAF · pcRAF + offset_pcRAF)
pMEK_obs	log10(scale_pMEK · (pMEK + ppMEK) + offset_pMEK)
pERK_obs	log10(scale_pERK · (pERK + ppERK) + offset_pERK)
pp70S6K_obs	log10(scale_pPS6K · pPS6K + offset_pPS6K)
tEGFR_obs	log10(scale_R1 · (R1 + 2 · R1R1 + R1R2 + R1R3 + R1R2_off) + offset_tR1)
tERBB2_obs	log10(scale_R2 · (R2 + R1R2 + R2R3 + R1R2_off + R2R3_off + 2 · R2R2_off) + offset_tR2)
tERBB3_obs	log10(scale_R3 · (R3 + R1R3 + R2R3 + R2R3_off) + offset_tR3)
tAKT_obs	log10(scale_AKT_HRG · (AKT + pAKT) + offset_tAKT_HRG)
tcRAF_obs	log10(scale_cRAF_HRG · (cRAF + pcRAF) + offset_tcRAF_HRG)
tMEK_obs	log10(scale_MEK · (MEK + pMEK + ppMEK)+ offset_tMEK)
tERK_obs	log10(scale_ERK · (ERK + pERK + ppERK) + offset_tERK)
t70S6K_obs	log10(scale_S6K · (S6K+pPS6K) + offset_tS6K)
R1_tot*	log10((R1 + 2 · R1R1 + R1R2 + R1R3 + R1R2_off)+8.44e-05)
R2_tot*	log10((R2 + R1R2 + R2R3 + R1R2_off + R2R3_off + 2 · R2R2_off)+8.44e-05)
R3_tot*	log10((R3 + R1R3 + R2R3 + R2R3_off)+6.96e-05)
AKT_tot*	log10((AKT + pAKT)+2.54e-05)
ERK_tot*	log10((ERK + pERK + ppERK)+ 2.14e-05)

Table S6. Estimated parameter values with confidence intervals. Parameter values of the global optimum for the ODE model calibrated on MCF7 and T47D cell lines along with profile-likelihood based confidence intervals all shown on logarithmic scale.

Parameter name	Estimated value (log10)	95%-confidence interval [log10(lower bound)]	95%-confidence interval [log10(upper bound)]
ActRatio_pcRAF_R1R1_EGF	3.24	3.01	3.48
ActRatio_pcRAF_R1R1_TGF	3.51	3.20	3.79
AKT_MCF7	-0.90	-0.94	-0.86
AKT_T47D	-1.58	-1.62	-1.53
build_R1R1_BTC	1.73	1.41	2.07
build_R1R1_EGF	1.04	0.84	1.25
build_R1R1_TGF	0.15	-0.09	0.38
build_R1R3_BTC	-2.88	-3.29	-0.93
build_R1R3_EGF	-2.94	-3.61	-1.62
build_R1R3_TGF	-0.98	-1.50	-0.72
cRAF_MCF7	-0.73	-0.97	-0.51
cRAF_T47D	-0.56	-0.76	-0.38
degrade_R1R3_BTC	-0.77	-1.27	1.29
degrade_R1R3_EGF	-1.01	-1.70	0.55
degrade_R1R3_HRG	-0.28	-0.57	0.13
degrade_R1R3_TGF	1.84	1.30	3.06
degrade_R2R3_HRG	-1.70	-1.93	-1.35
degrade_R3_lumretuzumab	-2.47	-2.50	-2.43
dephospho_pERK	-0.05	-0.23	0.27
dephospho_pMEK	0.00	-0.13	0.18
dephospho_S6K	-1.54	-1.62	-1.46
drug_R1R1_cetuximab	1.45	1.23	1.70
drug_R1R1_erlotinib	1.26	0.79	1.74
drug_R1R2_cetuximab	1.00	0.84	1.17
drug_R1R2_erlotinib	1.22	0.99	1.48
drug_R1R2_pertuzumab	-0.07	-0.22	0.06
drug_R1R2_trastuzumab	-0.32	-0.53	-0.15
drug_R1R3_erlotinib	0.45	0.14	0.81
drug_R2R3_erlotinib	0.01	-0.16	0.16
drug_R2R3_lumretuzumab	1.23	1.04	1.44
drug_R2R3_pertuzumab	1.15	1.01	1.31
drug_R2R3_trastuzumab	0.05	-0.11	0.19
drug_cRAF_R1R2_erlotinib	-0.06	-0.91	0.28
drug_cRAF_R2R3_erlotinib	0.68	0.34	1.33
ERK_MCF7	-2.90	-2.93	-2.86
ERK_T47D	-3.22	-3.26	-3.19
Kd_pAKT_basal	-1.99	-2.10	-1.88
Kd_pAKT_R1R2	1.96	1.70	2.33
Kd_pAKT_R2R3	2.05	1.80	2.42
Kd_pcRAF_basal	-1.23	-1.46	-1.04
Kd_pcRAF_R1R2	3.16	2.86	3.57
Kd_pcRAF_R1R3	2.93	2.61	3.36
Kd_pcRAF_R2R3	2.32	2.03	2.72
Kd_R1R2_BTC	1.38	0.95	1.68
Kd_R1R2_EGF	0.63	0.23	0.91
Kd_R1R2_TGF	0.32	-0.07	0.58
Kd_R1R2off_pertuzumab	0.22	-0.18	0.50
Kd_R1R2off_trastuzumab	0.60	0.21	0.86
Kd_R1R3_HRG	0.27	-0.14	0.57
Kd_R2R2off_pertuzumab	-0.31	-0.68	-0.07
Kd_R2R2off_trastuzumab	-0.29	-0.67	-0.04
Kd_R2R3_HRG	0.57	0.17	0.85
Kd_R2R3off_pertuzumab	-2.97	-3.13	-2.84
Kd_R2R3off_trastuzumab	-3.13	-3.34	-2.98

Parameter name	Estimated value (log10)	95%-confidence interval [log10(lower bound)]	95%-confidence interval [log10(upper bound)]
MEK_MCF7	-1.80	-2.03	-1.60
MEK_T47D	-1.46	-1.67	-1.31
mutation_T47D	-1.30	-1.39	-1.22
phospho_ERK	4.12	3.85	4.50
phospho_MEK	-0.40	-0.64	-0.14
phospho_MEK_basal	-2.61	-Inf	-2.23
phospho_S6K_AKT	0.21	0.08	0.34
phospho_S6K_ERK	2.76	2.56	3.01
R1_MCF7	-1.99	-2.02	-1.95
R1_T47D	-1.96	-2.00	-1.93
R2_MCF7	-1.74	-1.78	-1.71
R2_T47D	-1.34	-1.37	-1.30
R3_MCF7	-1.44	-1.47	-1.41
R3_T47D	-1.54	-1.56	-1.51
S6K_MCF7	0.48	0.47	0.50
S6K_T47D	-0.85	-0.90	-0.80
offset_pcRAF_BTC	-0.07	-0.10	-0.04
offset_pcRAF_EGF	-0.07	-0.09	-0.05
offset_pcRAF_HRG	-0.05	-0.08	-0.03
offset_pcRAF_TGF	-0.12	-0.16	-0.09
offset_pAkt_BTC	-0.31	-0.36	-0.27
offset_pAkt_EGF	-0.44	-0.51	-0.39
offset_pAkt_HRG	-0.83	-0.96	-0.73
offset_pAkt_TGF	-0.32	-0.38	-0.27
offset_pERK_EGF	-1.53	-2.42	-1.27
offset_pMEK_BTC	-0.28	-0.31	-0.25
offset_pMEK_EGF	-0.35	-0.37	-0.32
offset_pMEK_HRG	-0.41	-0.46	-0.37
offset_pMEK_TGF	-0.26	-0.29	-0.24
offset_pPS6K_BTC	-0.73	-0.87	-0.63
offset_pPS6K_EGF	-0.92	-1.16	-0.78
offset_pPS6K_TGF	-0.59	-0.69	-0.51
offset_pR1_BTC	-0.46	-0.49	-0.44
offset_pR1_EGF	-0.97	-1.00	-0.95
offset_pR1_HRG	-0.73	-0.76	-0.71
offset_pR1_TGF	-0.62	-0.64	-0.59
offset_pR2_BTC	-0.30	-0.31	-0.29
offset_pR2_EGF	-0.04	-0.05	-0.03
offset_pR2_HRG	-0.22	-0.23	-0.20
offset_pR2_TGF	-0.40	-0.41	-0.38
offset_pR3_BTC	-0.11	-0.12	-0.10
offset_pR3_EGF	-0.10	-0.11	-0.08
offset_pR3_HRG	-0.16	-0.18	-0.15
offset_pR3_TGF	-0.14	-0.15	-0.12
offset_tAKT_HRG	-0.15	-0.18	-0.13
offset_tcRAF_HRG	-0.07	-0.15	-0.02
offset_tERK_BTC	-0.16	-0.21	-0.12
offset_tERK_EGF	-0.11	-0.15	-0.07
offset_tERK_HRG	-0.15	-0.21	-0.11
offset_tERK_TGF	-0.15	-0.21	-0.11
offset_tMEK_BTC	-0.02	-0.03	-0.01
offset_tMEK_EGF	-0.07	-0.08	-0.06
offset_tMEK_HRG	-0.04	-0.05	-0.03
offset_tMEK_TGF	-0.05	-0.06	-0.04
offset_tR1_BTC	-0.89	-0.90	-0.87
offset_tR1_EGF	-0.99	-1.01	-0.97
offset_tR1_HRG	-0.92	-0.94	-0.91
offset_tR1_TGF	-0.99	-1.01	-0.98

Parameter name	Estimated value (log10)	95%-confidence interval [log10(lower bound)]	95%-confidence interval [log10(upper bound)]
offset_tR2_BTC	-0.51	-0.65	-0.41
offset_tR2_EGF	-0.55	-0.73	-0.44
offset_tR2_HRG	-0.76	-1.15	-0.58
offset_tR2_TGF	-0.55	-0.72	-0.44
offset_tR3_EGF	-0.73	-2.24	-0.44
offset_tR3_HRG	-0.17	-0.26	-0.10
offset_tS6K_BTC	-1.13	-1.32	-1.01
offset_tS6K_EGF	-1.05	-1.20	-0.95
offset_tS6K_HRG	-1.51	-2.50	-1.25
offset_tS6K_TGF	-1.26	-1.56	-1.10
scale_AKT_HRG	0.79	0.74	0.85
scale_pcRAF_BTC	0.82	0.62	1.03
scale_pcRAF_EGF	0.64	0.43	0.87
scale_pcRAF_HRG	0.73	0.51	0.95
scale_pcRAF_TGF	0.93	0.72	1.15
scale_ERK_BTC	2.61	2.52	2.71
scale_ERK_EGF	2.57	2.47	2.67
scale_ERK_HRG	2.64	2.55	2.74
scale_ERK_TGF	2.64	2.55	2.74
scale_pAkt_BTC	1.91	1.82	2.00
scale_pAkt_EGF	1.98	1.88	2.08
scale_pAkt_HRG	1.96	1.90	2.03
scale_pAkt_TGF	1.97	1.88	2.08
scale_pERK_BTC	3.76	3.68	3.86
scale_pERK_EGF	3.74	3.64	3.85
scale_pERK_HRG	3.92	3.83	4.02
scale_pERK_TGF	3.77	3.68	3.86
scale_pMEK_BTC	0.19	0.12	0.26
scale_pMEK_EGF	0.20	0.13	0.25
scale_pMEK_TGF	0.14	0.06	0.22
scale_pPS6K_BTC	0.63	0.54	0.72
scale_pPS6K_EGF	0.67	0.58	0.76
scale_pPS6K_HRG	0.61	0.53	0.70
scale_pPS6K_TGF	0.62	0.53	0.71
scale_pR1_BTC	2.27	2.04	2.62
scale_pR1_EGF	1.91	1.67	2.28
scale_pR1_HRG	2.01	1.77	2.37
scale_pR1_TGF	2.26	2.03	2.62
scale_pR2_BTC	2.50	2.29	2.83
scale_pR2_EGF	2.11	1.89	2.46
scale_pR2_HRG	2.11	1.91	2.45
scale_pR2_TGF	2.38	2.18	2.72
scale_pR3_EGF	1.24	0.64	1.54
scale_pR3_HRG	-0.92	-1.14	-0.58
scale_pR3_TGF	1.55	1.32	1.76
scale_R2_BTC	1.56	1.51	1.61
scale_R2_EGF	1.59	1.55	1.64
scale_R2_HRG	1.65	1.61	1.70
scale_R2_TGF	1.57	1.53	1.62
scale_R3_BTC	1.73	1.70	1.76
scale_R3_EGF	1.68	1.62	1.73
scale_R3_HRG	1.59	1.54	1.64
scale_R3_TGF	1.74	1.71	1.77
scale_S6K_EGF	0.00	-0.01	0.02
scale_S6K_HRG	0.02	0.01	0.04
scale_S6K_TGF	0.00	-0.01	0.02
sigma_EGFR	-1.10	-1.13	-1.07
sigma_ERBB2	-1.42	-1.45	-1.40

Parameter name	Estimated value (log10)	95%-confidence interval [log10(lower bound)]	95%-confidence interval [log10(upper bound)]
sigma_ERBB3	-1.35	-1.38	-1.32
sigma_pAKT_obs	-1.00	-1.02	-0.97
sigma_pcRAF_obs	-1.18	-1.21	-1.15
sigma_pEGFR_obs	-0.96	-0.98	-0.93
sigma_pERBB2_obs	-1.25	-1.28	-1.22
sigma_pERBB3_obs	-1.29	-1.32	-1.26
sigma_pERK_obs	-0.70	-0.73	-0.67
sigma_pMEK_obs	-1.14	-1.16	-1.11
sigma_pp70S6K_obs	-1.00	-1.02	-0.97
sigma_t70S6K_obs	-1.37	-1.40	-1.35
sigma_tAKT_obs	-1.51	-1.57	-1.46
sigma_tcRAF_obs	-1.48	-1.53	-1.42
sigma_tERK_obs	-1.39	-1.41	-1.36
sigma_tMEK_obs	-1.48	-1.51	-1.45

Table S7. Correlation coefficients of signaling features. The simulated signaling features tested for the linear proliferation model are listed along with their correlation coefficients in respect to the viability scores. Features for R2R3 were not calculated as this dimer is only formed upon NRG1 treatment, resulting in too little data points for the correlation analysis.

Feature	Coefficient
AUC_R1R1	0.44
AUC_R1R2	0.56
AUC_R1R3	-0.02
AUC_pAKT	0.79
AUC_ppERK	0.67
AUC_pPS6K	0.73
peak_R1R1	0.50
peak_R1R2	0.70
peak_R1R3	0.07
peak_pAKT	0.71
peak_ppERK	0.58
peak_pPS6K	0.62
slope_R1R1	0.50
slope_R1R2	0.67
slope_R1R3	0.30
slope_pAKT	0.38
slope_ppERK	0.20
slope_pPS6K	-0.08

Table S8. Parameters of the regression model.

Parameter name	Estimate	Std. Error	t value	p value
Intercept	0.917	0.007	138.646	$< 2e-16$
AUC_pAKT	0.080	0.0245	3.288	0.002
AUC_pPS6K	-0.009	0.023	-0.380	0.706

Table S9. Results of the L_1 analysis. Parameters fitted during the L_1 analysis are listed. Only indicated parameters were fitted with L_1 prior. Note, the parameters *PI3Kdiff_cellline* describe the difference in PI3K activity in relation to MCF7. MDA-MB-231 and SKBR3 are not mutated in PI3K, but have a different PI3K activity compared to MCF7.

Parameter name	Estimated value (log10)
**Protein totals and PI3K activity fitted without L_1 prior**	
AKT_MDA231	-1.53
AKT_SKBR3	-1.66
ERK_MDA231	-3.92
ERK_SKBR3	-2.88
MEK_MDA231	-1.09
MEK_SKBR3	-2.03
R1_MDA231	-1.06
R1_SKBR3	-1.31
R2_MDA231	-1.90
R2_SKBR3	0.15
R3_MDA231	-2.45
R3_SKBR3	-1.69
S6K_MDA231	-0.87
S6K_SKBR3	-0.06
cRAF_MDA231	-0.88
cRAF_SKBR3	-0.99
PI3Kdiff_MDA231	-0.38
PI3Kdiff_SKBR3	-1.95
**L_1 parameters fitted with L_1 prior**	
ActRatio_pcRAF_R1R1_L1_MDA231	0.03
ActRatio_pcRAF_R1R1_L1_SKBR3	0.00
Kd_R1R2_L1_MDA231	-1.08
Kd_R1R2_L1_SKBR3	-0.45
Kd_R1R2off_L1_MDA231	-1.14
Kd_R1R2off_L1_SKBR3	-1.94
Kd_R1R3_L1_MDA231	1.30
Kd_R1R3_L1_SKBR3	-1.58
Kd_R2R2off_L1_MDA231	-0.19
Kd_R2R2off_L1_SKBR3	-2.50
Kd_R2R3_L1_MDA231	0.00
Kd_R2R3_L1_SKBR3	-2.79
Kd_R2R3off_L1_MDA231	1.55
Kd_R2R3off_L1_SKBR3	0.00
Kd_pAKT_R1R1_L1_MDA231	0.00
Kd_pAKT_R1R1_L1_SKBR3	0.00
Kd_pAKT_R1R2_L1_MDA231	-0.34
Kd_pAKT_R1R2_L1_SKBR3	-0.32
Kd_pAKT_R1R3_L1_MDA231	0.00
Kd_pAKT_R1R3_L1_SKBR3	0.00
Kd_pAKT_R2R3_L1_MDA231	0.00
Kd_pAKT_R2R3_L1_SKBR3	0.98
Kd_pAKT_basal_L1_MDA231	0.35
Kd_pAKT_basal_L1_SKBR3	0.00
Kd_pcRAF_R1R2_L1_MDA231	-0.63
Kd_pcRAF_R1R2_L1_SKBR3	0.99
Kd_pcRAF_R1R3_L1_MDA231	-0.40
Kd_pcRAF_R1R3_L1_SKBR3	1.35
Kd_pcRAF_R2R3_L1_MDA231	0.00
Kd_pcRAF_R2R3_L1_SKBR3	1.66
Kd_pcRAF_basal_L1_MDA231	-1.65
Kd_pcRAF_basal_L1_SKBR3	-0.83
build_R1R1_L1_MDA231	-2.12

Parameter name	Estimated value (log10)
build_R1R1_L1_SKBR3	0.00
build_R1R3_L1_MDA231	-3.04
build_R1R3_L1_SKBR3	3.20
degrade_R1R1_L1_MDA231	0.00
degrade_R1R1_L1_SKBR3	0.00
degrade_R1R2_L1_MDA231	0.00
degrade_R1R2_L1_SKBR3	0.00
degrade_R1R3_L1_MDA231	0.77
degrade_R1R3_L1_SKBR3	0.58
degrade_R2R3_L1_MDA231	0.00
degrade_R2R3_L1_SKBR3	0.91
dephospho_S6K_L1_MDA231	0.65
dephospho_S6K_L1_SKBR3	0.50
dephospho_pERK_L1_MDA231	-0.78
dephospho_pERK_L1_SKBR3	0.00
dephospho_pMEK_L1_MDA231	0.27
dephospho_pMEK_L1_SKBR3	-0.01
phospho_ERK_L1_MDA231	0.09
phospho_ERK_L1_SKBR3	0.00
phospho_MEK_L1_MDA231	0.29
phospho_MEK_L1_SKBR3	0.00
phospho_MEK_basal_L1_MDA231	0.84
phospho_MEK_basal_L1_SKBR3	-0.83
phospho_S6K_AKT_L1_MDA231	0.00
phospho_S6K_AKT_L1_SKBR3	0.36
phospho_S6K_ERK_L1_MDA231	0.17
phospho_S6K_ERK_L1_SKBR3	0.00
**Offset and scaling parameters fitted without L1 prior**	
offset_pcRAF_SKBR3	-0.18
offset_pAkt_SKBR3	-0.46
offset_pERK_SKBR3	-0.56
offset_pMEK_SKBR3	-0.05
offset_pR1_SKBR3	-0.16
offset_pR2_SKBR3	-0.04
offset_pR3_SKBR3	-0.01
offset_pPS6K_SKBR3	-0.63
offset_tERK_SKBR3	-0.01
offset_tMEK_SKBR3	0.00
offset_tR1_SKBR3	-0.11
offset_tR2_SKBR3	-0.72
offset_tR3_SKBR3	-0.23
offset_tS6K_SKBR3	-0.38
scale_ERK_SKBR3	-0.45
scale_MEK_SKBR3	-0.45
scale_R1_SKBR3	0.83
scale_R2_SKBR3	-0.24
scale_R3_SKBR3	1.40
scale_S6K_SKBR3	-0.42
scale_pcRAF_SKBR3	0.71
scale_pAkt_SKBR3	1.29
scale_pERK_SKBR3	3.67
scale_pMEK_SKBR3	0.05
scale_pR1_SKBR3	1.88
scale_pR2_SKBR3	1.17
scale_pR3_SKBR3	-1.14
scale_pPS6K_SKBR3	-0.08

Data file S1. Viability screen of MCF7 and T47D cell lines.

Supplementary Methods

Model reduction

All models of complex biological systems are simplifications. However, to ensure reliable model predictions and thus provide a useful model, the model complexity should be tailored to the available information in the experimental data. Too complex models are more likely to overfit measurement errors and then provide inaccurate parameter estimates and predictions. In this project, this complexity adaptation of the ODE signaling model was realized by profile likelihood-based model reduction [2]. Therefor, the model was reduced until all parameters could be determined by a unique optimal value and finite confidence intervals, i.e. until all parameters were identifiable. In the following, the reduction of the ODE signaling model is exemplified by three major reduction steps that were performed.

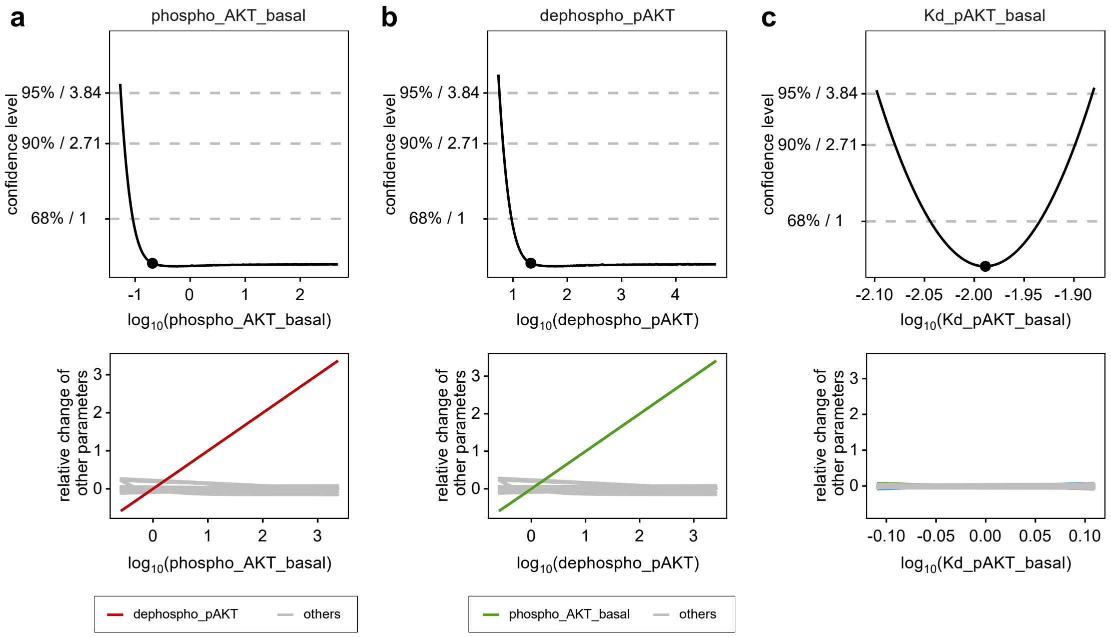

Figure SM1. Profile likelihood based model reduction of the AKT sub-module. The profile likelihood for (**a**) phospho_AKT_basal as well as (**b**) dephospho_pAKT are practically non-identifiable. (**c**) After the first model reduction step, a reparametrization, the ratio parameter Kd_pAKT_basal becomes identifiable.

In the first instance we regard two *practical non-identifiable* parameters [3] describing the basal phosphorylation (phospho_AKT_basal) and the dephosphorylation of AKT (dephospho_pAKT). As shown in Figure SM1a and b these two parameters are only bounded to lower values as the likelihood profile is flat towards large values. Obviously, the RPPA data used for the calibration of the signaling model does not provide information about the upper bound of these two parameters. However, as depicted in the lower panel of the plots, there exists a positive correlation between these two parameters. In this scenario, identifiability can be achieved by introducing the ratio parameter Kd_pAKT_basal $= \left(\frac{\text{phospho\_AKT\_basal}}{\text{dephospho\_pAKT}}\right)$. Replacing e.g. phospho_AKT_basal by Kd_pAKT_basal · dephospho_pAKT, the ratio parameter becomes identifiable and has no considerable dependencies to other parameters left (Figure SM1c). The remaining parameter dephospho_pAKT is flat and can be fixed to an arbitrary value.

A second scenario for model reduction is represented by the dynamic parameter *degrade_R1R2_TGF*, a model parameter that describes the degradation of the EGFR:ERBB2 hetero-dimer after TGF stimulation. In Figure SM1a the profile likelihood of this parameter is plotted along with its dependencies. Since the profile is flat towards small values and all

parameter dependencies are flat in this direction as well, this parameter is not necessary to describe the data, i.e. the degradation of the EGFR:ERBB2 dimer after TGF stimulation is not needed. Thus, this parameter can be fixed to zero retaining a good fit to the data.

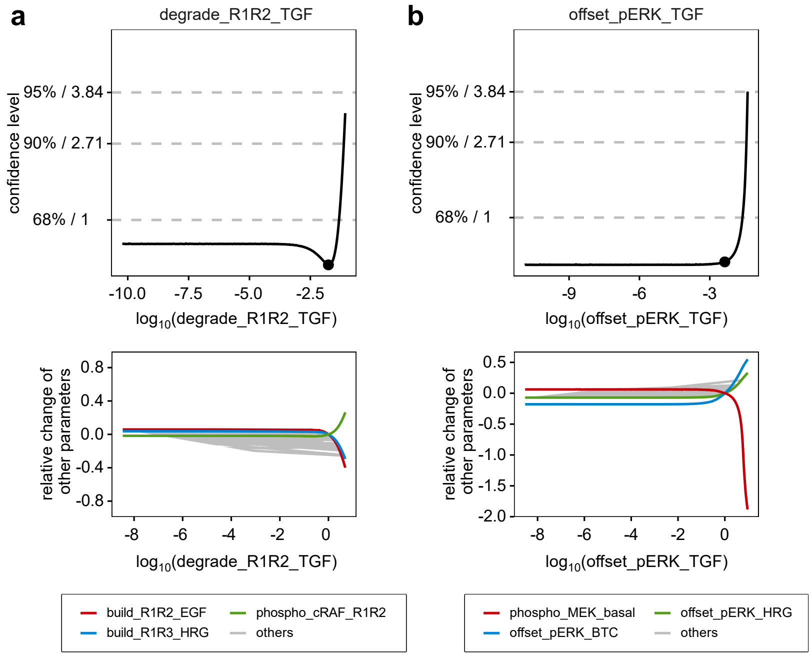

Figure SM2. Profile likelihood based model reduction of redundant parameters. The profile likelihood for (**a**) the dynamic parameter degrade_R1R2_TGF as well as (**b**) the observation-related parameter offset_pERK_TGF is flat towards small values, indicating that these parameters are not necessary for the data description. They can thus be fixed to zero.

Finally, the last scenario describes an observable-related model reduction step. The ODE signaling model presented in this work contains a lot of observable-related parameters as scalings and offsets in addition to dynamic parameters. Especially offset parameters are often dispensable, as most of our data is measured on relative scale. This is the case e.g. for the parameter *offset_pERK_TGF* that describes the offset for the observable pERK after TGF stimulation (Figure SM1b). This parameter is flat towards small values with flat dependencies and can be therefore removed, i.e. fixed to zero.

The here presented model reduction steps exemplify the reduction procedure which includes in total 63 model reduction steps. Even though a reduction step is usually dedicated to a specific parameter or parameter subset, the reduction might have an impact on other parameters. That is why the model was refitted after each reduction step and likelihood profiles were recalculated.

L_1 regularization for ODE models

Signal transduction is essential for all cells and is guided through a signal transduction network shared by most cell types. Even though pathway structures are mostly identical, kinetic rates or protein concentrations might differ between cell types originating, e.g. from mutations or gene amplifications. The identification of such differences is of major importance especially for cancer cells to evaluate treatment strategies. Such cell-specific differences can be analyzed with a mathematical model by testing which model parameters have to be considered as cell type-specific and which parameters can be treated as identical. A method to efficiently determine those parameters that have to be cell type-specific is the L_1 regularization [4]. This method was applied in our study to determine differences between the luminal cell lines T47D and MCF7 and (i) the TNBC MDA-MB-231 as well as (ii) the HER2-positive SKBR3 cell line.

To apply L_1 regularization, the ODE signaling model was reparametrized. Each dynamic model parameter p_2 of the analyzed cell line MDA-MB-231 or SKBR3 was expressed in relation to the corresponding parameter p_1 of the original luminal model as

$$p_2 = p_1 \cdot \Delta p \quad \text{or} \quad \log(p_2) = \log(p_1) + \log(\Delta p) \tag{1}$$

after transformation to the logarithmic scale. Here, Δp corresponds to the ratio between a dynamic parameter p_i in two cell lines. For $\log(\Delta p) = 0$ the parameters for the two cell lines are identical.

The concept of L_1 regularization is to add an L_1 penalty to the objective function, introduced in Equation 1 of the main paper, that penalizes exactly this ratio parameter Δp. Thus, the L_1 penalty forces the model to prefer solutions with equal parameters between the two cell lines. The new objective function yields

$$\chi^2(p, \Delta p) = \chi^2(p) + \lambda \left(\sum_i \left| \log(\Delta p_i) \right| \right) \tag{2}$$

with λ indicating the strength of the L_1 penalty. For the analysis of our additional cell lines we gradually increased the value of λ from 10^{-3} to 10^3, performing multi-start optimization for every λ.

The best value for the objective function $\chi^2(p, \Delta p)$ in Equation (2) will be achieved when $\lambda = 0$, as in this scenario all parameters can differ between the two cell lines, which gives the model the maximum freedom to describe the data. However, to select the best model with the optimal regularization strength λ, also the number of non-zero parameters has to be taken into account. Therefore, the AIC was calculated for each value of λ respectively and was used as selection criteria. Doing so, we could identify $\lambda = 10^{0.67}$ as the best regularization strength for the MDA-MB-231 cells as well as $\lambda = 10^{-0.65}$ for the SKBR3 cell line.

References

1. Niepel, M.; Hafner, M.; Pace, E.A.; Chung, M.; Chai, D.H.; Zhou, L.; Schoeberl, B.; Sorger, P.K. Profiles of Basal and stimulated receptor signaling networks predict drug response in breast cancer lines. *Sci. Signal.* **2013**, *6*, ra84. https://doi.org/10.1126/scisignal.2004379.
2. Maiwald, T.; Hass, H.; Steiert, B.; Vanlier, J.; Engesser, R.; Raue, A.; Kipkeew, F.; Bock, H.H.; Kaschek, D.; Kreutz, C.; et al. Driving the Model to Its Limit: Profile Likelihood Based Model Reduction. *PLOS ONE* **2016**, *11*, e0162366. https://doi.org/10.1371/journal.pone.0162366.
3. Raue, A.; Kreutz, C.; Maiwald, T.; Bachmann, J.; Schilling, M.; Klingmüller, U.; Timmer, J. Structural and practical identifiability analysis of partially observed dynamical models by exploiting the profile likelihood. *Bioinformatics* **2009**, *25*, 1923–1929. https://doi.org/10.1093/bioinformatics/btp358.
4. Steiert, B.; Timmer, J.; Kreutz, C. L1 regularization facilitates detection of cell type-specific parameters in dynamical systems. *Bioinformatics* **2016**, *32*, i718–i726. https://doi.org/10.1093/bioinformatics/btw461.
